# EGFR targeting monoclonal antibody combines with an mTOR inhibitor and potentiates tumor inhibition by acting on complementary signaling hubs

**DOI:** 10.1002/cam4.21

**Published:** 2012-08-01

**Authors:** Roshan James, Siddharth Vishwakarma, Indira V Chivukula, Chetana Basavaraj, Ramakrishnan Melarkode, Enrique Montero, Pradip Nair

**Affiliations:** 1 Biocon Ltd, R&D, Drug Development GroupBangalore, 560100, India; 2 Clinigene International LtdBangalore, 560100, India; 3 Center of Molecular ImmunologyHavana, 11600, Cuba

**Keywords:** Immunotherapy, Nimotuzumab, signal transduction, Sirolimus, synergy

## Abstract

Nimotuzumab, an anti-epidermal growth factor receptor (anti-EGFR) monoclonal antibody, has been used extensively in many solid tumors and confers significant survival advantage. The antibody has limited skin toxicity and is generally well tolerated. Similar to other anti-EGFR therapies, patients may relapse a few months after treatment. In this study we show for the first time, the use of Nimotuzumab along with Sirolimus has synergistic effect on tumor inhibition as compared with the drugs used individually, in Nimotuzumab responsive and nonresponsive cell lines. In vitro studies prove that while Sirolimus (25 nmol/L) affects the signal downstream to mammalian target of rapamycin (mTOR), Nimotuzumab (83 nmol/L) downregulates pTYR, pMAPK and pSTAT3 by 40%, 20% and 30%, respectively. The combination, targeting these two different signaling hubs, may be associated with the synergistic inhibition observed. In vivo, the use of half human therapeutic equivalent doses for both the drugs substantially reduces tumors established in nude as well as severe combined immunodeficiency (SCID) mice by EGFR overexpressing A-431 cells. The drug combination reduces cell proliferation and the expression of signal transduction molecules. Treated tumors are better differentiated as compared with those established in the control mice. Tumor microarray demonstrates that Nimotuzumab and the combination groups segregate independently to the Sirolimus and the control treatment. The combination uniquely downregulated 55% of the altered tumor genes, extending beyond the typical pathways associated with Nimotuzumab and Sirolimus downstream pathways inhibition. These results would suggest that this nontoxic drug combination improves therapeutic benefit even in patients with low-EGFR expression and severely immunocompromised because of their current medication.

## Introduction

Epidermal growth factor receptor (EGFR), a member of the HER family of receptor kinases is overexpressed in a wide range of tumor types including nonsmall cell lung, pancreatic, breast and head and neck cancers with several drugs targeting this molecule [1, 2, 3, 4]. Nimotuzumab also known as h-R3 or BIOMAb EGFR is a humanized anti-EGFR monoclonal antibody. Clinical trials and therapeutic use of this antibody, involving >7000 patients worldwide, have shown evidence of efficacy in the treatment of patients bearing advanced epithelial-derived tumors [5, 6]. Compared with other anti-EGFR therapies the low toxicity and the lack of skin rash with Nimotuzumab is an advantage [7]. At present, Nimotuzumab is approved for therapeutic use in cancer treatment in many countries including India [5]. The clinical effect with Nimotuzumab is observed when the drug was used alone or in combination with radiotherapy, chemotherapy or chemoradiation [6].

Sirolimus or Rapamycin is a lipophilic macrolide antibiotic originally isolated from a strain of the soil bacterium *Streptomyces hygroscopicus* [8, 9]. Sirolimus forms a complex with its intracellular receptor, the FK506-binding protein, FKBP12 which in turn binds a region in the C terminus of TOR proteins termed FRB thereby inhibiting TOR activity [10]. In the mammalian cell, mTOR-dependent processes involve regulating cell growth by controlling mRNA translation, ribosome biogenesis, autophagy and metabolism [11]. Over the years, two mTOR complexes have been identified, mTORC1 and mTORC2. While mTORC1 is sensitive, the mTORC2 complex is generally insensitive to Sirolimus [11, 12]. Effectors of mTORC1 include S6K1 and 4E-BP1 both regulators of mRNA translation. mTORC2 complexes with rapamycin-insensitive companion of mTOR (RICTOR) instead of regulatory associated protein of mTOR (RAPTOR) which then directly phosphorylates AKT at Serine 473 [13, 14]. This function positions mTOR at both sides of AKT [13, 14, 15]. Use of Sirolimus is associated with limited clinical success in oncology possibly because of the activation of AKT [11, 14].

Although a combination of EGFR targeting drugs and Sirolimus has been tried before [16], we show for the first time that the monoclonal antibody targeting EGFR namely Nimotuzumab in combination with Sirolimus has a synergistic inhibitory effect on epithelial cells. In vivo, the suboptimal human therapeutic equivalent doses of drugs in combination, showed more tumor reduction than the drugs used individually and this is associated with the downregulation of critical signal transduction molecules including pMAPK, pSTAT3 and PCNA along with better tumor differentiation. In addition, the sustained inhibition observed in vivo with pAKT with the combination of drugs proved that the presence of Nimotuzumab prevented the feedback activation of pAKT by Sirolimus [14]. While combinatorial therapies have been used extensively to control carcinoma [17], in this study we demonstrate proof of concept for the use of Sirolimus and Nimotuzumab as combination therapy. We believe that the low toxicity of Nimotuzumab associated with its lower affinity makes it more agreeable for this strategy.

## Materials and Methods

### Cell lines

The cell line A-431, ATCC® CRL-1555™ an epidermoid carcinoma cell line was maintained in Dulbecco's Modified Eagle's Medium (DMEM) supplemented with 1% Penicillin–Streptomycin, 20 mmol/L 4-(2-hydroxyethyl)-1-piperazineethanesulfonic acid (HEPES) and 10% Fetal Bovine Serum (FBS). BxPC-3, ATCC® CRL-1687™ a pancreatic adenocarcinoma cell line was maintained in Roswell Park Memorial Institute (RPMI)-1640, 1% Penicillin–Streptomycin and 10% FBS.

### Cell authentication

The A-431, ATCC® CRL-1555™ (Sourced from ATCC), BxPC-3, ATCC® CRL-1687™ (Sourced from ATCC). A working cell bank was made from this ATCC sourced vial and this was tested at ATCC for DNA profile (Short Tandem repeats) and confirmed to be identical to the parent cell lines. Routine evaluation of morphology along with Mycoplasma contamination testing (PCR Mycoplasma Test Kit II, PromoKine, Heidelberg, Germany and Hoechst 33258 staining) was performed for both the cell lines. Receptor densities (EGFR) quantified regularly with Spherotech (Spherotech, Inc., Lake Forest, IL) fluorescent beads were routinely performed [18].

### Reagents

Sirolimus manufactured in house was reconstituted in 1 mL dimethyl sulfoxide (DMSO) to obtain a final concentration of 12.2 mmol/L and subsequently reconstituted in media for the in vitro as well in vivo assays. Nimotuzumab (BIOMAb EGFR) is manufactured at Biocon Limited at a concentration of 5 mg/mL.

### Sulforhodamine B (SRB) colorimetric assay

The percentage inhibition of proliferation of A-431 and BxPC-3 cells were calculated using SRB assay as described by Skehan et al. [19], [20].

The A-431 human epidermoid carcinoma cells were seeded at a concentration of 5000 cells per well and were incubated in a humidified CO_2_ incubator at 37°C for 24 h. One plate was fixed using 10% trichloro acetic acid (TCA) to be used as the 0-h baseline value. Different dilutions of the drugs alone or in combination were added to the cells and incubated for 72 h in a humidified CO_2_ incubator at 37°C. The spent medium was tapped off from each plate and the cells were fixed using 10% TCA. After fixation, all plates were stained using 0.4% SRB in 1% acetic acid, washed with 1% acetic acid, eluted using 10 mmol/L unbuffered Tris base and read at 570 nm in an enzyme-linked immunosorbent assay (ELISA) plate reader. The percentage inhibition of the A-431 cells was calculated with respect to the untreated after 72 h.

### Quantification of EGFR receptors on cell surface

Both the cells were treated with the same concentration range of Nimotuzumab for 1 h at 4°C. The cells were then washed using phosphate buffered saline (PBS; SIGMA D8537). The fluorescein isothiocyanate (FITC)-conjugated secondary antibody (SIGMA F9512) was added for 30 min. The cells were resuspended in PBS and acquired using Flowcytometer (Beckman Coulter, Fullerton, CA, USA). The receptors were then quantified as described previously [18].

### Analysis of additivity and synergy

The Bliss Independence model was used to classify the effect of combining Sirolimus and Nimotuzumab as additive, synergistic or antagonistic [1]. A theoretical Bliss curve was calculated for combined cytotoxicity, that is a curve representing the additive cytotoxic effect of the two drugs, using the equation E_bliss_ = E_A_ + E_B_ − (E_A_ × E_B_), where E_A_ and E_B_ are the fractional cytotoxicities obtained by drug A alone and drug B alone at specific concentrations. The Bliss Analysis plot was generated using Prism GraphPad software version 5.

### Preparation of protein lysates and western blotting

Cell extracts were prepared by radioimmunoprecipitation assay (RIPA) buffer containing phenyl methyl sulfonyl fluoride (PMSF), protease inhibitor cocktail and sodium orthovanadate (sc-24948, Santa Cruz Biotechnology, Santa Cruz, CA). The primary antibodies included phospho-Tyrosine [#9411] (pTYR), phospho-Akt (S473) [#9271] (pAKT), Akt pan11E7 [#4685], phospho-MAPK (p42/p44) [#9101] (pMAPK) and phospho-Stat3 (Y705) [#9131] (pSTAT3), phospho-S6 Ribosomal Protein (S235/236) [#2211] (pS6RP). The primary antibodies were obtained from Cell Signaling Technologies (Danvers, MA). TFIID (TBP) (SI-1) [sc-273] from Santa Cruz Biotechnology was used as a loading control. T1h, an anti-CD6 monoclonal antibody was used as an isotype control.

### Study of drug combination in a sc-tumor xenograft model

Animal studies were approved by the animal ethical committees. Five million cells were injected in BALB/c nude mice in a single s.c. site on the left flank. Tumors were allowed to grow to at least 200 mm^3^, at which time the animals were sorted into treatment groups of six animals per group based on even distribution of body weight tumor volumes and clinical observations were measured daily and body weights were determined weekly. The tumor volume was determined by measuring with vernier calipers and calculated using the following formula: tumor volume = 4/3π (radius^3^), with radius determined using averaged length and width measurements. Six doses were administered over a 2-week period, given by i.p. injection using an insulin syringe. Half human equivalent dose was calculated by multiplying the human dose with 12.3 as per pharmacological guidelines (Guidance for Industry, FDA, July 2005). Nimotuzumab was introduced first, followed by Sirolimus 1 h later. All control animals were dosed with equal volumes of the vehicles. Treatment combinations were as follows: Placebo, 12.5 nmol/L Sirolimus, 606.5 nmol/L Nimotuzumab and 606.5 nmol/L Nimotuzumab + 12.5 nmol/L Sirolimus. After the last dosing on day 28, all mice were sacrificed after 2 h of dosing.

The C57BL6/severe combined immunodeficiency (SCID) Mice were 8–10 weeks old and sourced from Jackson laboratories, USA. Doses were identical to that given in the BALB/c nude mice bred at Center for Cellular and Molecular Biology (CCMB). Seven animals per group were used. Dosing was given from day 7 till day 18. A total of six doses were given at a similar dose and in an identical fashion as done for the BALB/c nude mice. Tumors extracted were collected in RNA later followed by processing in the histopathology laboratory where it arrived within an hour and was cut into two halves where one half went for immunohistochemistry and the other was sent for RNA extraction and subsequent microarray analysis.

### Hematoxylin and eosin and immunohistochemistry staining on paraffin-embedded formalin-fixed tumor tissue

Half of tissue extracted from the SCID mice in RNA later was subsequently formalin-fixed processed and embedded in wax. Microtome sections at five micron thick were taken on poly l-lysine coated slides. Routine hematoxylin and eosin (H and E) staining was performed as described previously [2]. Immunohistochemistry was performed on tissue sections as described earlier [21]. The primary antibodies used were phospho-Akt (S473) [#9271] (pAKT), phospho-MAPK (p42/p44) [#9101] (pMAPK) and phospho-Stat3 (Y705) [#9131] (pSTAT3) all from Cell Signaling Technologies, proliferating cell nuclear antigen (PCNA) (PC 10) from Sigma – Aldrich, USA. The color development was performed using VECTASTAIN Elite ABC system (VECTOR LABORATORIES, INC, Burlingame, CA). All the staining was performed on 1 day.

One of the two evaluators was a pathologist. Photomicrographs were taken using an inverted Nikon TE2000 S microscope with CCD camera, evolution VF from Media cybernetics and Image pro plus software version 6.01. Three independent 20× fields were captured and number of positive brown cells in each field counted. Percent positive cells were calculated using the formula (number of cells positive/total number of cells × 100). The intensity of expression was also documented. However, as all the slides showed moderate expression in a scale of low, moderate, high intensity [2], the staining intensity was not considered and percentage staining alone was reported.

### Microarray analysis

Processing of tissue was as described previously [18]. Genotypic designed Custom Whole Genome Human 8 × 60K (AMADID No: 027114) used (Agilent Technologies, Agilent, Palo Alto, CA, USA). Differentially regulated genes were clustered using hierarchical clustering based on Pearson's coefficient correlation algorithm to identify significant gene expression patterns genes were classified based on functional category and pathways using GeneSpringGX Software and Genotypic Biointerpreter**-**Biological Analysis Software. The accession number for microarray data is GSE32333, http://www.ncbi.nlm.nih.gov/geo/query/acc.cgi?acc=GSE32333.

### Validation of microarray by RT-PCR

To validate the microarray data, 1500 ng of DNase-treated RNA was reverse transcribed to make 50 ng/*µ*L of complementary DNA (cDNA) using Affinity Script qPCR cDNA synthesis kit (Agilent – Lot# 6077352). Relative quantification by qPCR was then done using Brilliant II SYBR Green qPCR Master mix (Lot # 1105284). Each sample was run in duplicates for each gene using 50 ng input per reaction. The experiment was conducted using Stratagene Mx3005P (Agilent Technologies) platform. The relative expression levels of the genes were determined after normalizing with MRPS30 as the reference gene by using ΔCt method. Eight genes involved in the mTOR and EGFR signaling pathway which also showed significant difference in microarray from the control ranging from moderately significant to highly significant (*P*-value < 0.05–0.000) were chosen for validating the microarray data.

### Statistical analysis

All analysis was based using the graph pad software version 5 statistical tools.

## Results

### The combination of Nimotuzumab with Sirolimus enhances inhibition of EGFR-expressing cells as compared with the individual drugs alone

**Figure 1:**
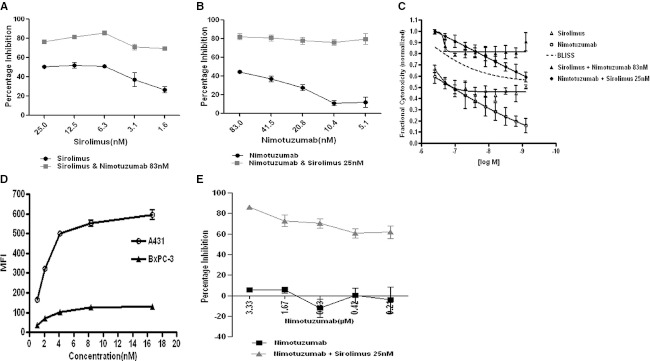
The combination of Nimotuzumab with Sirolimus enhances inhibition of EGFR-expressing cells as compared with the individual drugs alone. (A) Sirolimus shows a dose-dependent inhibition of proliferation in A-431 cells using SRB assay. In combination with a fixed concentration of Nimotuzumab (83 nmol/L), the level of inhibition is sustained across the Sirolimus range (25–1.56 nmol/L). (B) Nimotuzumab shows a dose-dependent inhibition across the concentration range 83–5.1 nmol/L. In combination with a fixed concentration of Sirolimus (25 nmol/L), the level of inhibition is sustained even at lower concentrations of Nimotuzumab. The gray line across each data point is significantly different from the dark line (*t*-test analysis). (C) The combination of Nimotuzumab and Sirolimus on A-431 cells is synergistic. A theoretical bliss curve (dotted line in the figure) demonstrating additive response was calculated for combined cytotoxicity. The experimental curves obtained with varying concentrations of Sirolimus (400–0.78 nmol/L) and fixed concentration of Nimotuzumab (83 nmol/L) and with varying concentration of Nimotuzumab (400–0.78 nmol/L) and a fixed concentration of Sirolimus (25 nmol/L) lie above the theoretical additive curve suggesting synergistic inhibition. The x-axis shows the log-transformed values of drug concentration in molar (mol/L). (D) Quantification of the EGFR receptor on A-431 and BxPC-3 cells was done using a flow cytometer Beckman Coulter Cyan ADP. Anti-EGFR antibody Nimotuzumab was used at a similar concentration range in both the cell lines. A-431 shows at least fourfold higher mean fluorescence intensity as compared with BxPC-3 cells. (E) Nimotuzumab (3.33–0.05 *μ*mol/L) with a fixed concentration of Sirolimus (25 nmol/L) shows much higher cytotoxicity as compared with the drug alone. The threshold of Sirolimus seems to be important in these cells, as lower concentration of Sirolimus with fixed amount of Nimotuzumab has around 20% cytotoxic effect (Fig. S1). Figure represents one of the four independent experiments. Error bars are standard error around the mean in all the figures.

With Nimotuzumab (83 nmol/L) and Sirolimus (25 nmol/L) alone the maximum inhibition observed was close to 50% (Fig. 1A and B). Interestingly, at low concentration of Sirolimus 1.6 nmol/L with a fixed concentration of Nimotuzumab 83 nmol/L, the inhibition observed was close to 70%. Similarly, at low concentration of Nimotuzumab (5.2 nmol/L) with Sirolimus of 25 nmol/L the inhibition observed was close to 80%. These two results suggested that the combination of the drugs had more inhibitory potential than either of the drugs used alone. The graphs are representative of multiple independent experiments (*n* = 4) performed on A-431 cells using SRB assay.

To understand whether the combination of the drugs was additive or synergistic a Bliss analysis was performed on A-431 cells. As shown in Figure 1C, the drugs used in combination had greater inhibition than the theoretical additive curve (BLISS) generated (dotted line) when the effect of the two drugs is considered additive. Sirolimus and Nimotuzumab at varying concentrations were used to calculate the additive curve. In both cases the effect of the combination was synergistic. The Bliss graph shown is a representative of two independent experiments performed.

The BxPC-3, another EGFR-expressing cell line with much lower receptor density (Fig. 1D and Table 1) as compared with A-431 has no inhibition with Nimotuzumab even at 3.33 *μ*mol/L concentrations (Fig. 1E). Sirolimus at 25 nmol/L with varying Nimotuzumab has high cytotoxicity (60–100% from *n* = 4 experiments) while Sirolimus alone at 25 nmol/L has low, around 20% toxicity (Fig. S1). This shows that even in this pancreatic low EGFR-expressing cell line, the combination is more effective in inhibiting tumor cell proliferation as compared with the drugs alone.

**Table 1:** Receptor density calculated using Spherotech beads from the Figure 1D shows that A-431 cells has got four times receptor when compared with BxPC-3 cells

Cell line	Concentration (nmol/L)	MFI	Receptor density (×10^6^/cell)
A431	16.67	595	0.67
BxPC-3	16.67	130	0.15

The table shows concentration used, mean fluorescence intensity (MFI) at the maximum dose and the receptor density of both the cells.

### Combination affects the cell proliferation by targeting different signal transduction hubs

**Figure 2:**
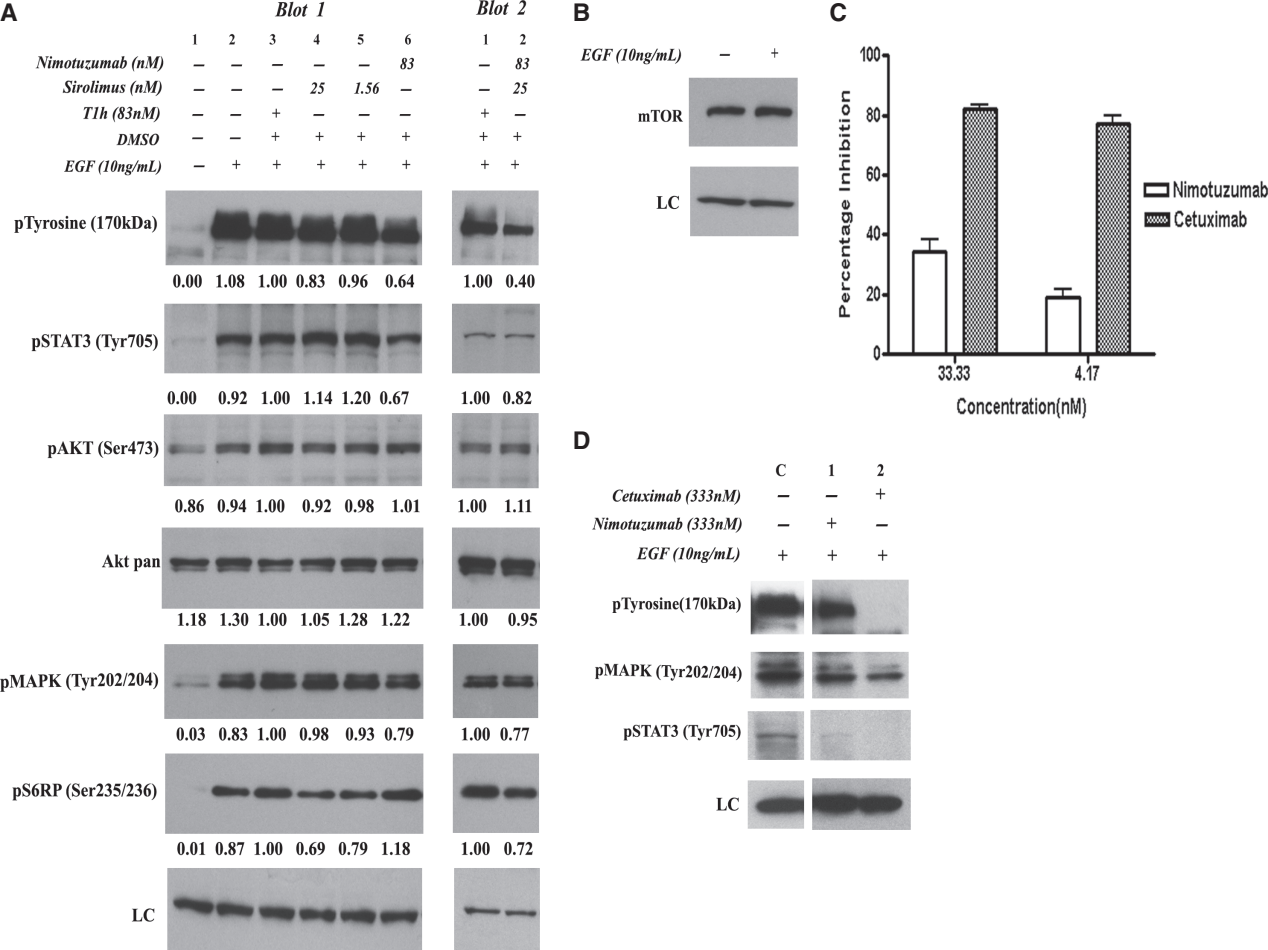
Nimotuzumab and Sirolimus in combination independently downregulate signaling proteins downstream to EGFR and mTOR. (A) One million A-431 cells were seeded in a 6-well plate and incubated for 24 h with the drugs alone or in combination in presence of 1% serum. The cells were then spiked with 10 ng/mL of EGF for 10 min. The cell lysates were prepared and the total protein was estimated using Bradford's method. The proteins were then separated by 10% sodium dodecyl sulfate polyacrylamide gel electrophoresis (SDS-PAGE), transferred overnight to the polyvinylidene fluoride (PVDF) membrane. The expressions of various proteins were checked by standard Western blot analysis. The blots were developed by enhanced chemiluminescence as per the manufacturer's protocol. Two blots were used and the expression is normalized to their respective controls in the blots. The numbers below the blot are relative to the respective controls from arbitrary values generated from the Alpha view software. (B) mTOR expression is high in A-431 cells unstimulated or stimulated with EGF. (C) Cetuximab showed higher inhibition of proliferation of A-431 cells at equimolar concentrations when compared with Nimotuzumab using SRB assay. Error bars are SD around the mean. (D) Nimotuzumab and Cetuximab were incubated for 2 h at 333 nmol/L and in the presence of EGF at 10 ng/mL for 10 min. Cetuximab at these equimolar concentrations is a better inhibitor of EGFR downstream signaling molecules. Lane C is a control treated with EGF and a polyclonal irrelevant antibody. This was run on the same blot at a different lane from lanes 1 and 2 (Fig. S2).

We hypothesized that the synergy of Nimotuzumab with Sirolimus observed in the cell inhibition assay might be attributed to the downregulation of independent signaling proteins from the two signal transduction pathways, mTOR and EGFR. So a Western blot analysis was performed and probed for specific signal transduction molecules. Relative expression of various proteins downstream to Nimotuzumab and Sirolimus are as depicted in Figure 2A. Two blots were used for the study – blot 1 and blot 2; both had separate assay controls. While blot 1 shows that Nimotuzumab at 83 nmol/L (12.5 *μ*g/mL) is able to downregulate pTYR (40%), pMAPK (20%) and pSTAT3 (30%) it has no effect under the current conditions to inhibit pAKT. Nimotuzumab at this concentration also does not inhibit pS6RP which is downstream to the mTOR signal transduction pathway. Sirolimus is able to reduce the pS6RP in a dose-dependent manner as shown in Figure 2A by 30% and 20% respectively for 25 and 1.56 nmol/L of Sirolimus. Sirolimus at 25 nmol/L has modest effect on pTYR expression by around 17% and this is shown as an additive effect with Nimotuzumab in blot 2 (Fig. 2A). Although the combination as shown in Figure 2A, blot 2 does not show synergistic inhibition for the signal transduction molecules, the phenotypic synergism observed in Figure 1A–C may be attributed to the fact that the drugs target different signal transductions hubs independently. Figure 2B would suggest that the basal expression of mTOR protein is high in A-431 cells and the expression does not alter when stimulated with EGF for 10 min. While both Nimotuzumab and Cetuximab are inhibitors of the EGFR signal transduction pathways, we observe significant difference in their activity as shown in Figure 2C and D. While Nimotuzumab at 33.3 nmol/L inhibits by 34%, Cetuximab at the same concentration shows 81% inhibition which is sustained by Cetuximab even at 4.17 nmol/L but not by Nimotuzumab (Fig. 2C). At equimolar concentrations, Cetuximab is a more potent inhibitor of the signal transduction molecules downstream to EGFR than Nimotuzumab (Fig. 2D). Full blots shown in Figure S2.

### In vivo studies in two different tumor models proved the effect of combination

**Figure 3:**
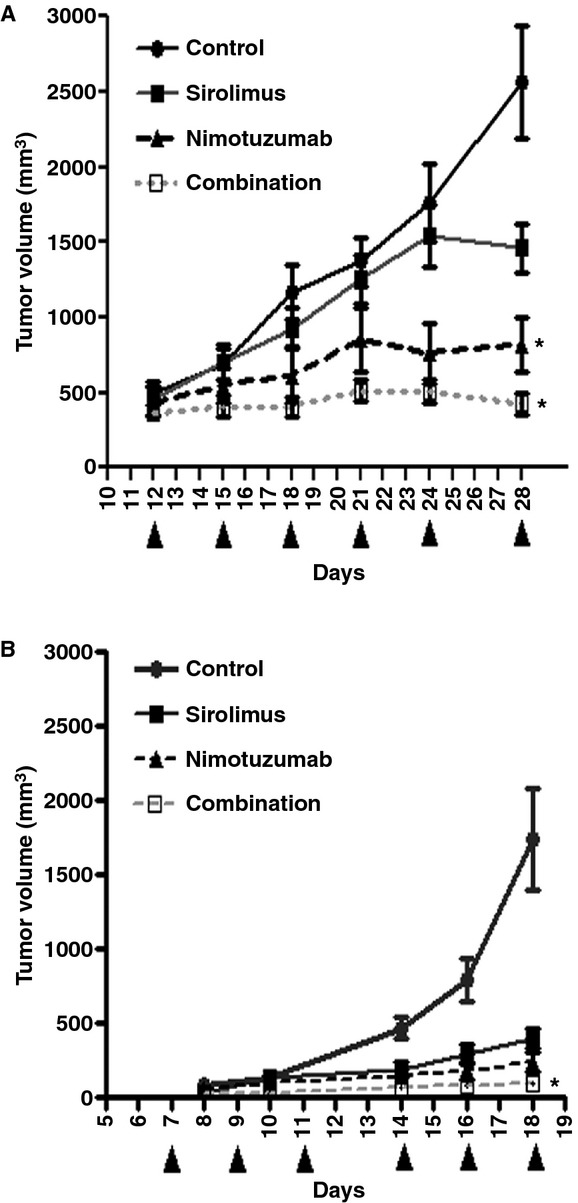
Significant reduction in tumor volume using Nimotuzumab and even better with the combination in different tumor models. (A) BALB/c nude mice carrying xenografts of A-431 cells respond significantly to Nimotuzumab but not to Sirolimus. However, the combination inhibits better than Nimotuzumab alone. Stars indicate significance using the Friedman's test followed by Dunn's multiple comparison tests. (B) A-431 cells (5 × 10^6^ cells/dose) were injected in SCID mice and the dosing were identical to that in the BALB/c nude mice except that the dosing was started earlier on day 7 without the tumors attaining 200 mm^3^ as in (A). The mice were then sacrificed on day 18. Graphs show standard error around the mean.

The A-431 cells were injected into C57BL/6-SCID mice (lacking B and T cells). There were seven animals in each group. This study followed the initial study performed in BALB/c nude (lacking T cells) mice (Fig. 3A) where the dosing is as shown in Figure 3A. The amount of drug dosed in mice (Placebo, 12.5 nmol/L Sirolimus, 606.5 nmol/L Nimotuzumab, and 606.5 nmol/L Nimotuzumab + 12.5 nmol/L Sirolimus) was estimated from the current therapeutic human equivalent doses 3.33 mg/kg/week for Nimotuzumab [5] and 0.42 mg/kg/week [22] for Sirolimus. The hypothesis being that at these suboptimal concentrations of the drugs the combination would perform better in these in vivo models substantiating the study performed earlier in vitro. Friedman's test followed by Dunn's multiple comparison showed significance only for the Nimotuzumab and the combination arms in these nude mice with the combination being more significant. In the SCID mice, we decided to dose the same amount but increase the frequency of dosing and curtail the experiment at an earlier time point as tumor biopsy samples extracted from the earlier nude mice study showed large areas of necrosis thereby confounding the immunohistochemistry results (data not shown). In addition, we wanted to mimic a scenario in vivo, where both T and B cells are compromised because of extensive chemotherapy as is observed with some patients. In this experiment all three arms showed significant reduction in tumor volume as compared with the control as measured by analysis of variance (ANOVA) analyzed on day 18 (*P*-value < 0.0001). However, Friedman's test followed by Dunn's multiple comparison showed significance only for the combination arm (Fig. 3B). This would suggest that the combination behaved better in two independent animal models. The aim in these animal models was not to show synergism, in the relatively short duration of the study, but to show better efficacy of the drugs in combination over the individual drugs alone. No significant difference in tumor metastasis into different organs could be observed visually in the different groups in the time scale of the studies.

### Tumor section evaluation demonstrates the effect of the combination

**Figure 4:**
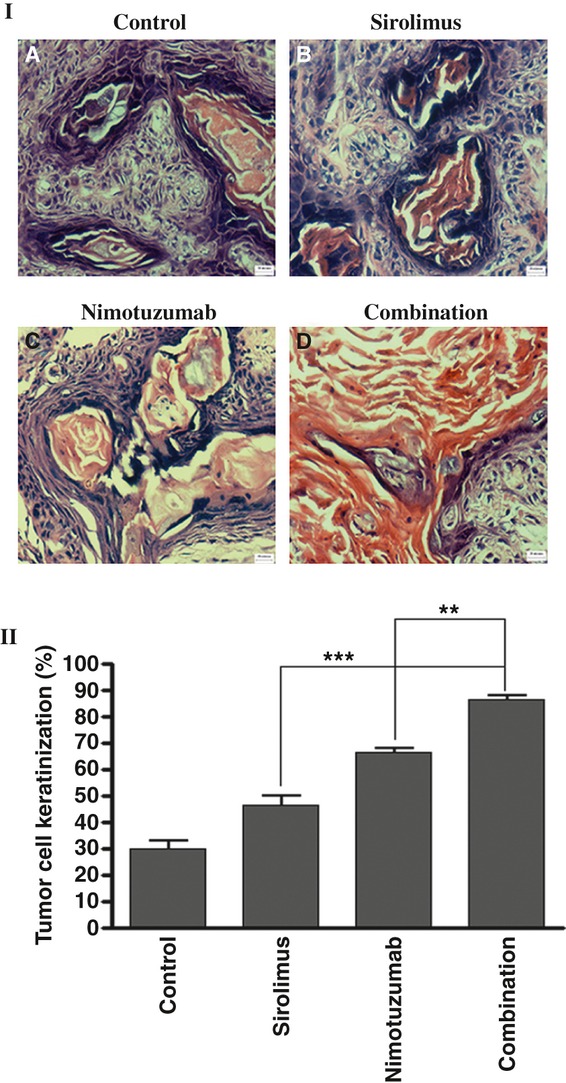
Combination therapy shows more differentiation within the tumors as shown by H and E evaluation. Five-micrometer-thick sections after formalin fixation and wax embedding were taken and stained using standard H and E techniques. Four animals from each group were evaluated for keratinization the animals were based on the two larger and smaller tumors from each group. (I) The control and the Sirolimus groups behave similarly with less differentiation and less keratinization. The amount of differentiation increases in the Nimotuzumab-treated groups while with the combination there is most differentiation with large areas of keratinization. (II) Depicts the amount of keratinization as a percent of the total tumor section. Each bar is an average of four sections from four different animals evaluated in each group. All the groups show significant keratinization as compared with the control. However, the amount of keratinization was maximum in the combination group. Error bars are standard deviation around the mean in all the figures.

**Figure 5:**
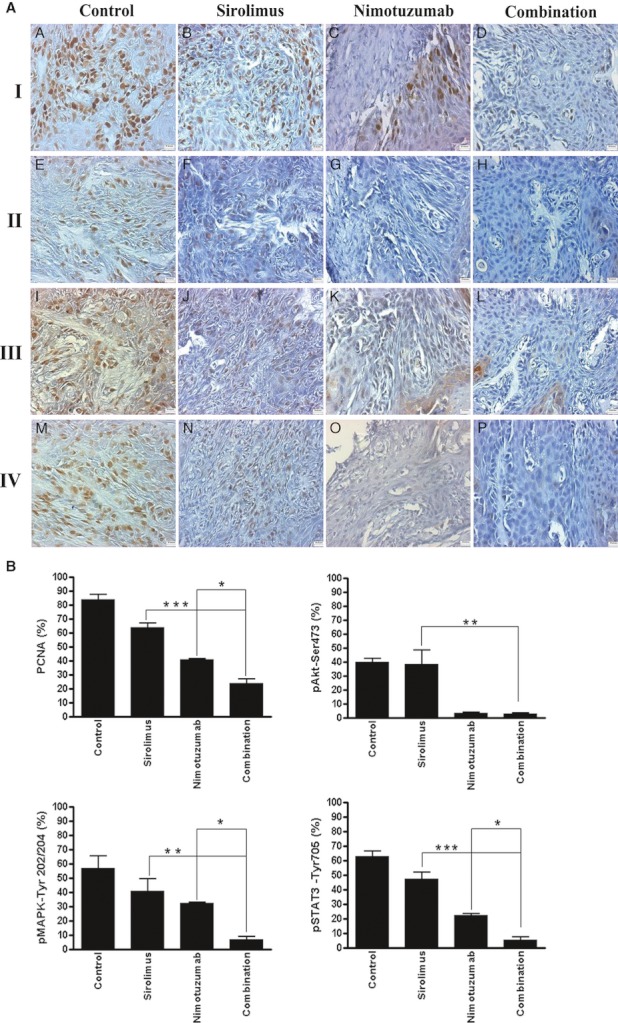
Nimotuzumab and Sirolimus in combination downregulates various signaling proteins downstream to EGFR as shown by immunohistochemistry. (A) Five-micrometer-thick sections were taken and stained using standard immunohistochemistry techniques from formalin-fixed and wax-embedded tumor sections. Primary antibodies were incubated for 2 h followed by washes in buffer and developed using a secondary antibody developing kit from Vectastain. Panel I (A–D) shows the expression of PCNA in tumor sections. Panel II (E–H) shows the expression of pAKT, Panel III (I–L) shows the expression of pMAPK while panel IV (M–P) depicts pSTAT3 expression. (B) Shows the percent cells positive as measured from three independent 20× photomicrographs taken from the microscope. Error bars show standard deviation. *P < 0.05, **P < 0.01, ***P = 0.001, respectively, relative to control by ANOVA. Error bars are standard deviation around the mean in all the figures.

H and E staining was performed on paraffin-embedded blocks from tumors obtained from SCID mice. There was less differentiation with very small areas of keratinization observed in the control and Sirolimus group as compared with Nimotuzumab and the combination groups. The combination showed more differentiated tumor with 80–90% of the total section scanned, showing areas of keratinization (Fig. 4I and II) with a significant difference over the Nimotuzumab and Sirolimus arms. Well-differentiated tumors closely resemble the tissue of origin and tend to grow slowly where as poorly differentiated or undifferentiated tumors reflect tumor progression and they grow quickly and have a tendency to spread. The combination therapy showed that the tumors were well differentiated. This suggests that the combination therapy resulted in inhibition of tumor progression which is also reflected by the reduced expression of the proliferation markers PCNA in these tissues. The decrease in PCNA, pMAPK and pSTAT3 was significant in the combination group as compared with the drugs alone (Fig. 5A and B). With pAKT, inhibition is observed with Nimotuzumab and is sustained in the combination group. This would suggest that the feedback activation of pAKT by Sirolimus [14] is blocked in the presence of Nimotuzumab in the combination group.

### Microarray analysis of tumor tissue

**Figure 6:**
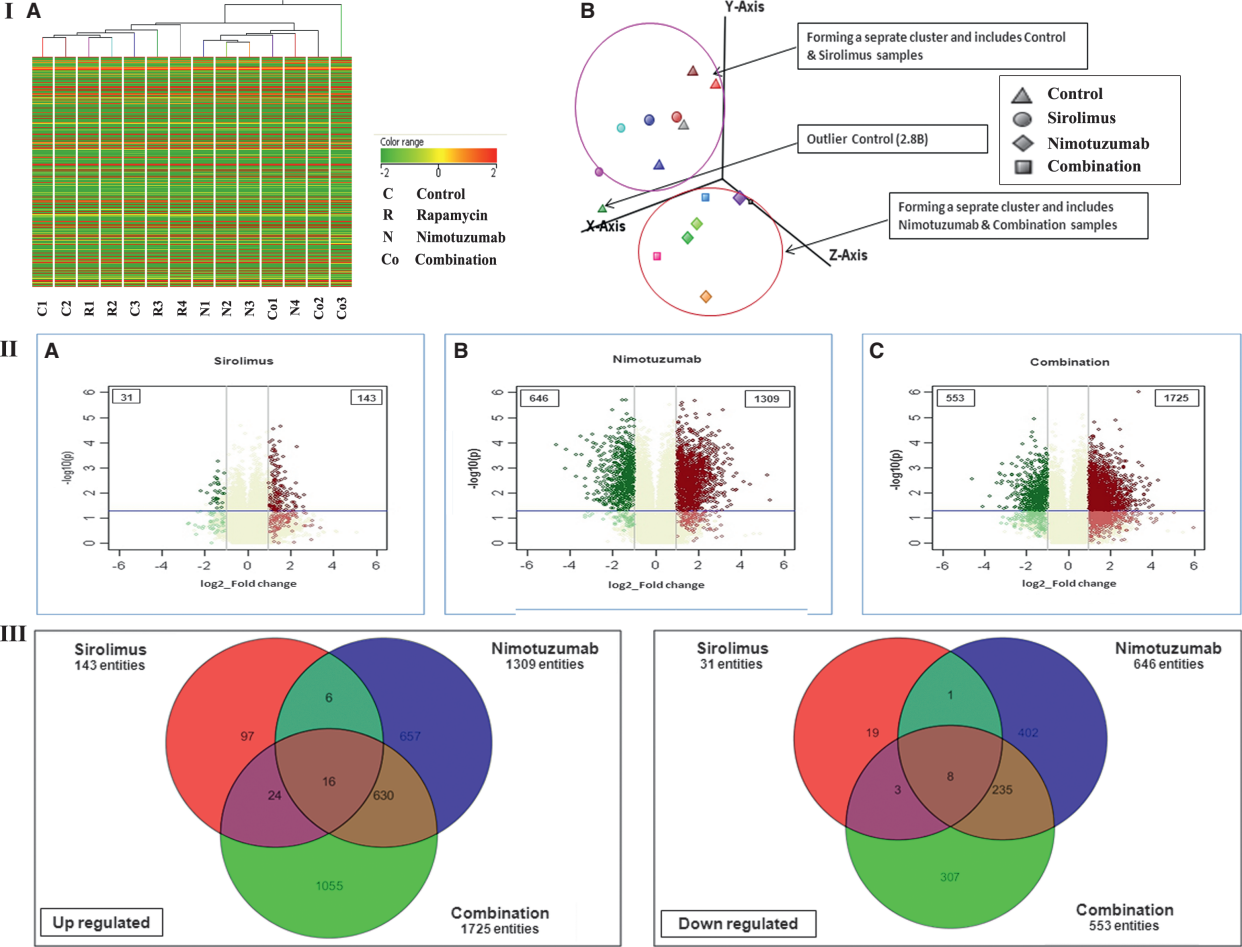
Tumors from combination therapy cluster differently in a microarray analysis compared with Sirolimus and Nimotuzumab group. (IA) Interarray clustering after removing the outlier control sample 2.8B showed that the clusters were spontaneously formed based on the treatment received. While the controls and the Sirolimus-treated groups were clustered together, the Nimotuzumab and the combination group clustered separately. One from the combination group was eliminated as RNA quantity obtained was insufficient. (IB) Principle component analysis shows that the Sirolimus and the control group are clustered together as compared with the Nimotuzumab and the combination group. Outlier 2.8B is shown. (IIA–C) The volcano plots show the expression of genes relative to the control. These graphs are generated using the R program. This plot shows the negative log 10 of *P*-value versus log of fold change. The horizontal purple line distinguishes significant *P*-value genes (above purple line). The genes falling in between two vertical lines are those having fold change value <−1 to >1. While the upper right quadrant shows significant with fold >1 and *P*-value <0.05 the upper left quadrant significant with fold >1 and *P*-value <0.05. The figures inside the graph show the total number of genes which are significantly downregulated or upregulated relative to the control biopsies (left side down and right side is upregulated). (III) Venn diagrams show the degree of similarity in the treated groups.

To avoid bias, two larger and the two smaller tumors from each group (from the SCID mice study) were evaluated for microarray analysis. The 2.8B sample defined as an outlier by principle component analysis was subsequently removed from the analysis (this sample was the largest and slightly necrotic; Fig. 6IB). The samples were then clustered spontaneously into two groups as shown in Figure 6IA and IB. Figure 6IIA–C show the volcano plots which describe the total number of genes which are significantly different from the control. Sirolimus is almost identical to the control, however, Nimotuzumab and the combination show a larger set of genes which are significantly up or downregulated. The significant differentially regulated genes relative to the control in the Sirolimus group were 143 upregulated and 31 downregulated, in the Nimotuzumab group are 1309 genes upregulated and 646 downregulated and the combination group are 1725 upregulated and 553 downregulated (Fig. 6II). Figure 6III shows that 61% and 55.5% of the genes are uniquely upregulated or downregulated respectively in the combination group. Additional data show a significant deregulation of signal transduction, metabolic pathway genes, cell adhesion molecules in the combination group from Biointerpreter pathway analysis (Genotypic) is as shown in Table S1A and B.

The 60K array was evaluated specifically for genes involved in mTOR and EGFR signal transduction using Biointerpreter pathway analysis software. The maximum number of genes upregulated significantly in these pathways are 4, 89 and 99 for the Sirolimus, Nimotuzumab and combination groups while the genes downregulated significantly were 3, 44 and 34 respectively. Among these genes, eight genes were validated using RT-PCR (these genes showed significant differences to control varying from moderately significant to highly significant). Data obtained from RT-PCR and microarray are comparable (Fig. S3 and Table S2).

## Discussion

Nimotuzumab has been used extensively in many clinical trials involving solid epithelial tumors [23]. The drug has been used in conjunction with radiation and chemoradiation and shown to have clinical efficacy in several studies [24]. The present study demonstrates the improved efficacy of Nimotuzumab by combining with a macrolide Sirolimus which affects another signal transduction hub within the malignant cells.

The signaling pathways that regulate mTOR activity are frequently activated in human cancers [25, 26, 27, 28]. In our studies we observe that the A-431 cells are positive for mTOR (Fig. 2B). In addition, these cells are sensitive to mTOR inhibitor-Sirolimus as shown by pS6RP protein downregulation (Fig. 2A). There is strong evidence that like yeast TOR, mTOR is required for cell progression and inhibition of mTOR activity by Sirolimus arrests cells in the G1 phase of the cell cycle. S6K and eLF4E, decreasing the translation of proteins like cyclin D1 and c-MYC and increasing apoptosis are involved in this process [29, 30, 31, 32].

The use of Sirolimus, which is a potent inhibitor of mTORC1 has had modest and unpredictable successes in oncology clinical trials possibly because mTOR reassembled in the mTORC2 rictor complex phosphorylates and activates AKT, thereby putting AKT on both sides of the signaling hub [11]. This activated AKT could then signal tumor activity inherently because of the pleiotropic nature of the molecule [33]. A-431 cells over express EGFR and use this pathway to signal as shown in Figure 2 wherein addition of ligand EGF spontaneously stimulates the cells. This is identical to results from other studies [34, 35]. Preincubation with an antibody Nimotuzumab which competes with EGF for the ligand-binding domain of EGFR causes a modest downregulation of the signal transduction as measured by pTYR by around 40% and pMAPK by around 20% while pSTAT3 is decreased by around 30% (Fig. 2A). EGF can bind with different affinities to EGFR varying 5–20 fold [36] while Nimotuzumab has an affinity constant of around 4.5 × 10^−8^ mol/L [37]. Hence, this may explain why in our hands preincubating the drugs and then following up with EGF for 10 min yielded the best results. Nimotuzumab has no effect in the signal transduction downstream to mTOR in this experimental setup. The effect on pAKT by Nimotuzumab is more in the long-term as observed in the in vivo experiments wherein Nimotuzumab as well as the combination is able to inhibit the expression of pAKT (Fig. 5A and B) but not in the short time of 10 min in the in vitro experiment. This would suggest that the cumulative effect of doses in vivo with Nimotuzumab is able to affect pAKT expression. The nonreduction of pAKT in the western blot may also be associated with the fact that even a few molecules of EGF can activate the receptor in the presence of Nimotuzumab, an antibody with reduced affinity for EGFR and this is sufficient to activate pAKT. This, we believe, differentiates Nimotuzumab from the other monoclonals, like Cetuximab and Panitumumab and other small molecule tyrosine kinase inhibitors having much higher affinities and consequently more significant skin toxicities [38, 39]. Differences between Cetuximab and Nimotuzumab have been reported before [38, 40, 41]. Nimotuzumab by itself has been shown previously to have profound effects in vivo animal models as described previously [35, 42]. The aim of this study in vivo was not to identify synergism in the relatively short duration of study, but to see whether combination could lead to an even more significant reduction in the volume of tumors and affect signal transduction more profoundly in these animal models. In addition, the duplication of the experiments in nude and SCID mice (Fig. 3A and B) mimic a scenario in patients where, T cells or both T and B cells are affected in patients undergoing chemotherapy. Although previous studies showed that in vitro, cells bound by Nimotuzumab do not exhibit an apoptotic phenotype, in vivo treated tumors on the other hand, display a fivefold increase in apoptotic activity generating a marked tumor regression [42]. In our study we did not observe enhanced cytotoxicity in vitro as the combination never showed a value lesser than the cells alone control in both SRB (Fig. 1A, B and E) as well as 3-(4,5-dimethylthiazol-2-yl)-5-(3-carboxymethoxyphenyl)-2-(4-sulfophenyl)-2H-tetrazolium assays (MTS) (data not shown) suggesting cell inhibition rather than cell depletion because of cytotoxicity. Multiple mechanisms can contribute to tumor regression in vivo which include decrease in vascular endothelial growth factor (VEGF) [42, 43], signal transduction inhibition, G0/G1 cell cycle arrest [37], apoptosis and antibody-dependent cell-mediated cytotoxicity (ADCC) [37]. A blend of these factors may be involved in the reduced tumors seen in the combination group in our study and this needs to be evaluated further. In this study, analysis of tumors in the in vivo experiment shows that the Sirolimus group has similar expression of pAKT as that of the control with some areas showing enhanced amount of pAKT (higher SD) as shown in Figure 5A and B. This may suggest that in the long-term in vivo, the mTORC2 pathway is activated and this in turn causes the enhanced phosphorylation of AKT as described previously [12, 14]. The targeting of the EGFR signal transduction upstream to AKT in vivo by Nimotuzumab may explain the reduced pAKT while the sustained inhibition with the combination may suggest that the mTORC2-mediated phosphorylation of AKT at S473 is not possible with the combination of these drugs. Analysis of the tissue biopsies from the xenograft SCID mice model would suggest that the combination can not only block the tumor cells from dividing as noted from the reduced expression of PCNA (Fig. 5A) but also has a role to play in preventing the loss of differentiation of the tumor (Fig. 4I and II). Tissue microarray clearly demonstrated the differences between the groups. A diverse set of genes as represented in Table S1A and B and Figure 6 are significantly different from the control in this combination group. The large number of genes uniquely upregulated and downregulated would suggest that the combination in vivo has an effect much beyond the typical pathways associated with signal transduction downstream to Nimotuzumab and Sirolimus. The number of upregulated genes may be indicative of feedback pathways causing genes to be reactivated (Figure 6III and Table S1B). Further evaluation of genes involved in the Sirolimus, Nimotuzumab and combination groups is ongoing. In a cell line expressing low levels of EGFR receptor (Fig. 1D), the combination of Sirolimus and Nimotuzumab is highly synergistic although the effect of Sirolimus is modest while Nimotuzumab has no effect in these cells (Fig. 1E). This result is similar to a study performed earlier wherein in an Erlotinib nonsensitive cell line only when Erlotinib was combined with Sirolimus, was synergistic inhibition observed [1].

Nimotuzumab has also been used extensively in many indications including lung cancer, pediatric glioma and works effectively in head and neck cancers overexpressing the receptor [2, 44, 45, 46]. While most anti-EGFR molecules are associated with high skin toxicity probably because of the high affinity of these molecules to EGFR expressed on the skin, Nimotuzumab with lower affinity does not demonstrate this [7]. Here we show that, Nimotuzumab has more subtle effects on signal transduction as compared with Cetuximab (Fig. 2C and D) as reported before [40]. Although in this study, Cetuximab has not been used along with Sirolimus, we believe that Nimotuzumab is more amenable to this combination because of its inherent lower toxicity. In this study, there is a possibility of suboptimal concentrations of Nimotuzumab and Sirolimus evaluated, being effective against solid tumors as demonstrated by the use of half human therapeutic equivalent dose of the drugs. In addition, data from the BxPC-3 cells supports the fact that a cell line expressing much lower EGFR receptors (four times less) as compared with A-431 may still be susceptible to a combination of Nimotuzumab and Sirolimus (Fig. 1D and E). However, this would need to be evaluated further. The present study demonstrates a proof of concept whereby the combination of Nimotuzumab and Sirolimus may have therapeutic advantage at lower therapeutic doses. This would indicate a potential for the use of the combination chronically, with the added benefit of the possibility of the drugs working in both high and low EGFR-expressing positive tumors.

## Supporting Information

**Figure S1.** Inhibition of proliferation using BxPC-3 cells wherein Nimotuzumab and Sirolimus alone are used along with Nimotuzumab varying and Sirolimus constant and vice versa. The assay showed that Nimotuzumab at all concentrations with a fixed concentration of Sirolimus at 25 nmol/L shows much higher cytotoxicity as compared with the drugs alone. The threshold of Sirolimus seems to be important in these cells, as lower concentration of Sirolimus with fixed amount of Nimotuzumab has not much cytotoxic effect. Figure represents one of the four independent experiments. Error bars are standard deviation around the mean.

**Figure S2.** Full blots showing the blots shown in Figure 2D in the manuscript.

**Figure S3.** Validation of microarray data. Figure 3A and B show similarity with gene expression profile obtained from RT-PCR and from microarray data. The actual values are shown in the corresponding tables. For RT-PCR, the Ct values determined were subtracted from the control MRPS30 values to obtain ΔCt (DCT) values. The DCT values were averaged for each group (Sirolimus, Nimotuzumab and combination). The ΔΔCt (DDCT) was calculated by subtracting DCT of the control group from the target or treated group. 2∧ (DDCT) was then performed to obtain absolute values. Absolute values are converted to log base2 for comparison with microarray data.

**Table S1A and B.** The tables list genes showing significant difference as seen from a pathway analysis software called biointerpreter developed at Genotypic. Signal transduction, metabolic pathway genes, cell adhesion molecules, and other pathways are affected in the combination group. Table 1A lists the gene families that are downregulated in the combination group while Table 1B lists the gene families that are upregulated in the same group. The numbers in the bracket are the total number of genes involved in the pathway in this array.

**Table S2.** The table lists the genes validated by RT-PCR confirming microarray data.
