# Supplementary figures and images for: EGFR targeting monoclonal antibody combines with an mTOR inhibitor and potentiates tumor inhibition by acting on complementary signaling hubs
Source: Cancer Med. 2012 Aug 1;1(2):114–27. doi: 10.1002/cam4.21 (PMC3544456; doi:10.1002/cam4.21)

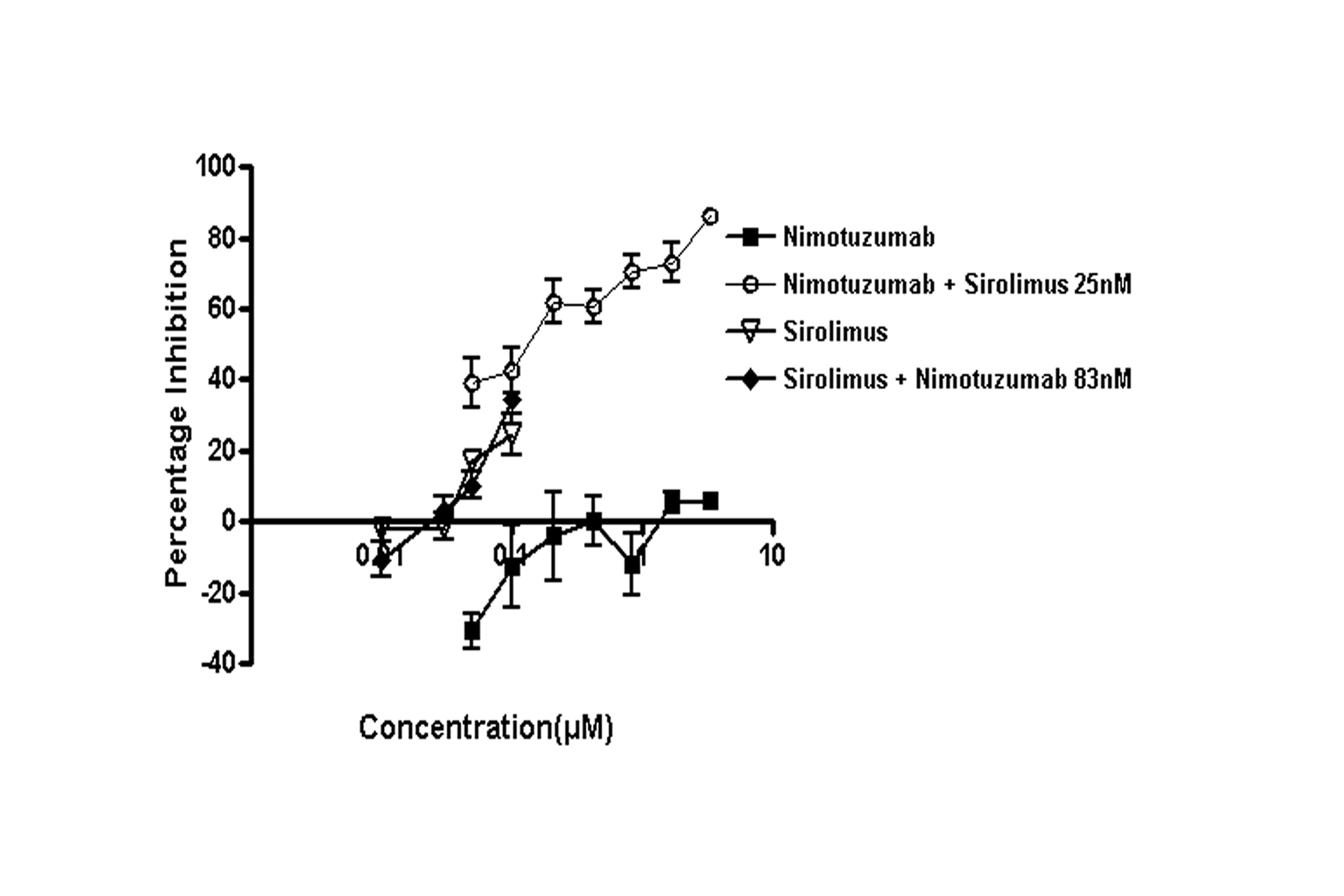

Supplement: Supplementary file 1 [file cam40001-0114-SD1.tif]

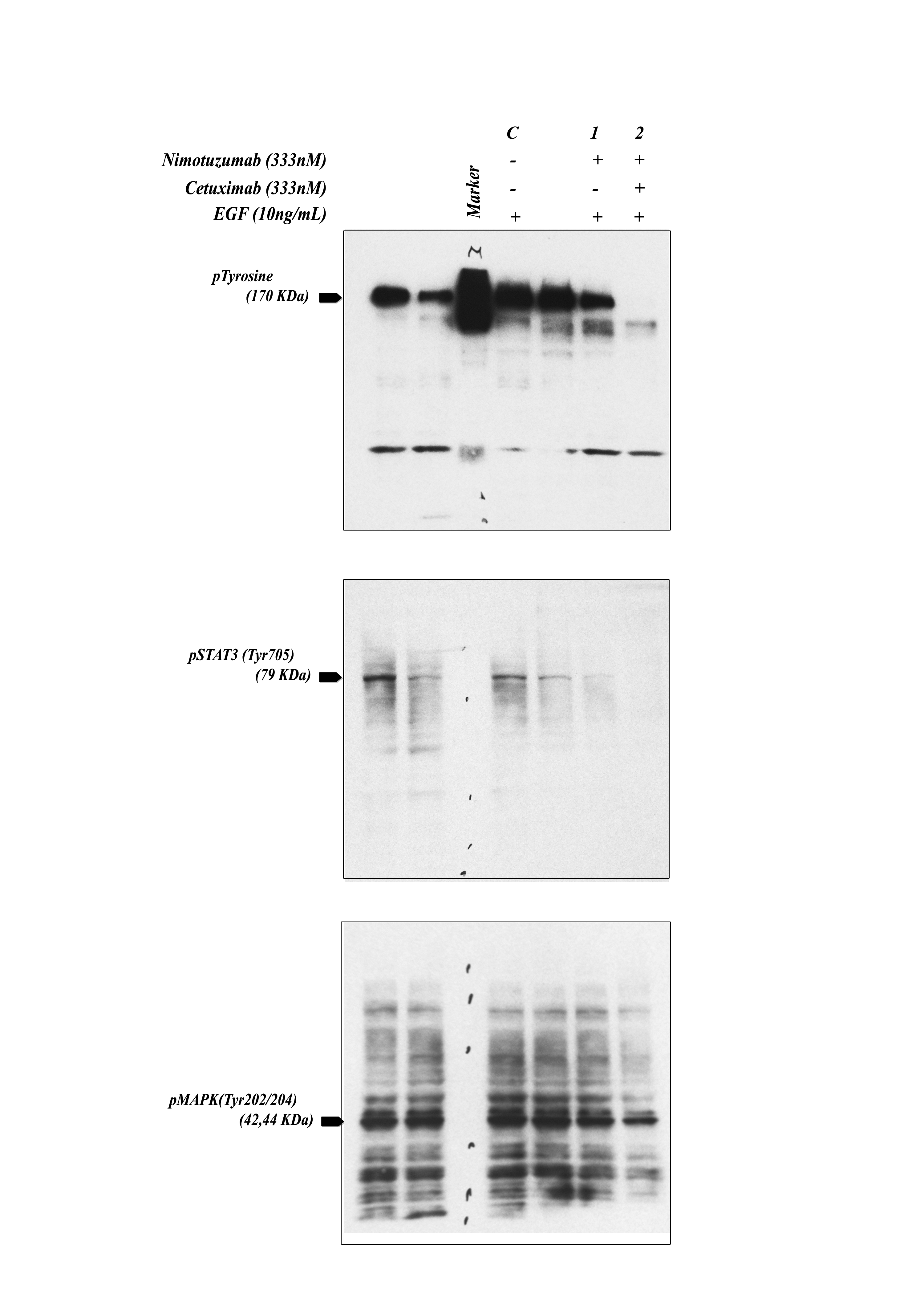

Supplement: Supplementary file 2 [file cam40001-0114-SD2.tif]

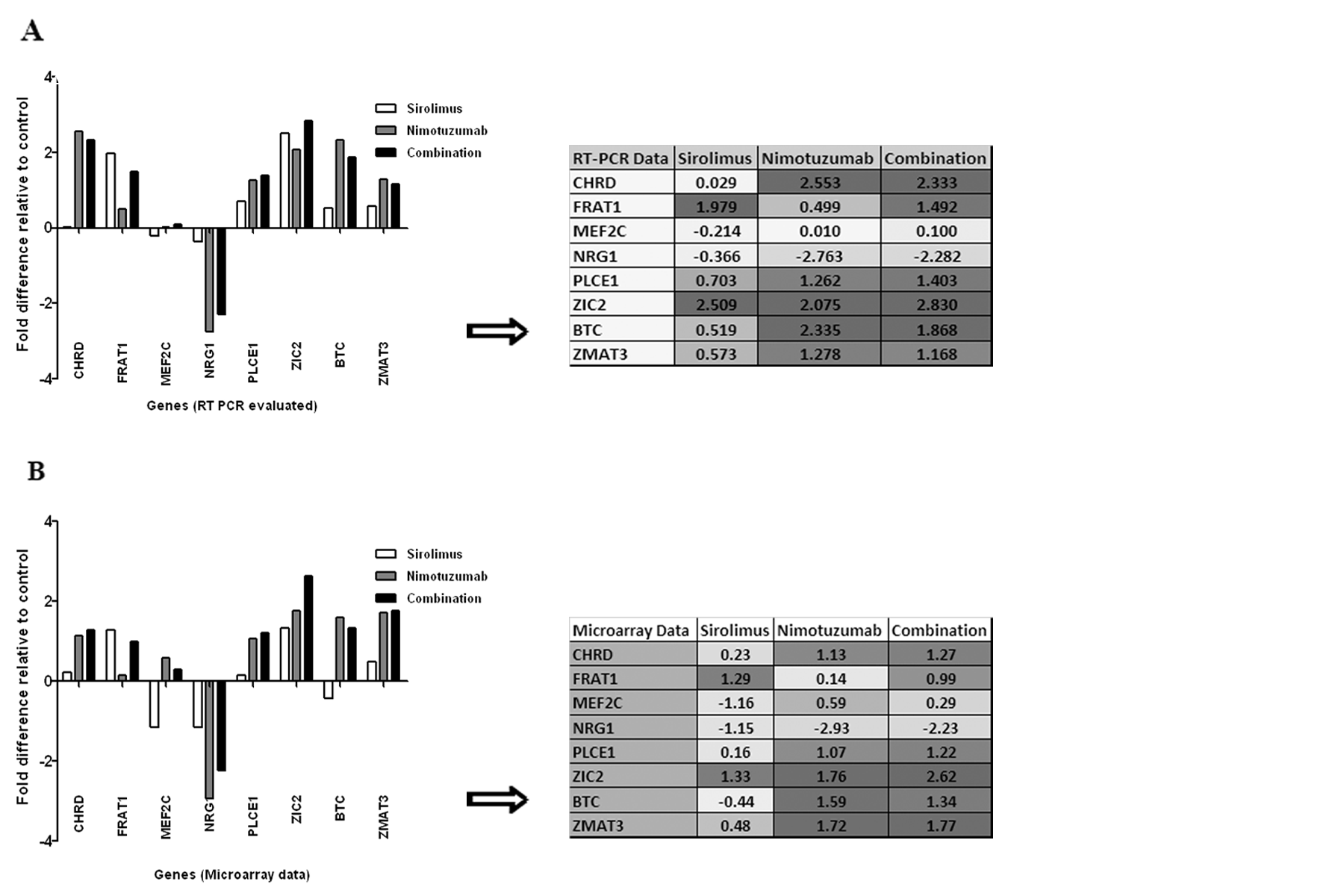

Supplement: Supplementary file 3 [file cam40001-0114-SD3.tif]
