# Supplementary material for: EGFR targeting monoclonal antibody combines with an mTOR inhibitor and potentiates tumor inhibition by acting on complementary signaling hubs
Source: Cancer Med. 2012 Aug 1;1(2):114–27. doi: 10.1002/cam4.21 (PMC3544456; doi:10.1002/cam4.21)
Supplement: Supplementary file 4 [file cam40001-0114-SD4.doc]

**Table 1A: Genes Down regulated**

| **Gene pathway** | **Number of genes** | **p Value** |
| --- | --- | --- |
| Metabolic pathways[1084] | 27 | 1.00E-09 |
| Pathogenic Escherichia coli infection[61] | 3 | 1.00E-09 |
| ECM-receptor interaction[86] | 11 | 1.00E-09 |
| Biosynthesis of unsaturated fatty acids[24] | 3 | 1.00E-09 |
| Cardiac muscle contraction[74] | 2 | 1.00E-09 |
| p53 signaling pathway[70] | 3 | 1.00E-09 |
| Antigen processing and presentation[80] | 2 | 1.00E-09 |
| Graft-versus-host disease[37] | 1 | 1.00E-09 |
| Pancreatic cancer[73] | 2 | 1.00E-09 |
| DNA replication[37] | 1 | 1.00E-09 |
| Methane metabolism[7] | 1 | 1.00E-09 |
| SNARE interactions in vesicular transport[35] | 1 | 1.00E-09 |
| Cytokine-cytokine receptor interaction[276] | 9 | 0.0186 |
| Pathways in cancer[341] | 13 | 0.0255 |
| Ubiquitin mediated proteolysis[139] | 3 | 0.0316 |
| Tight junction[131] | 3 | 0.0426 |

**Table 1B: Genes Up regulated**

| **Gene pathway** | **Number of genes** | **p Value** |
| --- | --- | --- |
| Olfactory transduction[390] | 20 | 1.00E-09 |
| Asthma[30] | 5 | 1.00E-09 |
| Pathways in cancer[341] | 18 | 1.00E-09 |
| Hypertrophic cardiomyopathy (HCM)[91] | 7 | 1.00E-09 |
| Cell adhesion molecules (CAMs)[134] | 9 | 1.00E-09 |
| Fc epsilon RI signaling pathway[81] | 3 | 1.00E-09 |
| Neuroactive ligand-receptor interaction[312] | 13 | 2.00E-04 |
| MAPK signaling pathway[276] | 13 | 1.30E-03 |
| Systemic lupus erythematosus[139] | 5 | 7.80E-03 |
| Vascular smooth muscle contraction[129] | 5 | 1.37E-02 |
| Calcium signaling pathway[189] | 10 | 1.62E-02 |
| Focal adhesion[201] | 11 | 1.62E-02 |
| Jak-STAT signaling pathway[159] | 8 | 0.0216 |
| Regulation of actin cytoskeleton[216] | 13 | 0.0243 |
| Cytokine-cytokine receptor interaction[276] | 19 | 0.0337 |
| Natural killer cell mediated cytotoxicity[135] | 7 | 0.0395 |

**Table 2: List of genes validated by RT-PCR confirming microarray data.**

| **Genes** | **Pathways** |
| --- | --- |
| CHRD | hsa04350:TGF-beta signaling pathway |
| FRAT1 | hsa04310:Wnt signaling pathway |
| MEF2C | hsa04010:MAPK signaling pathway |
| NRG1 | hsa04012:ErbB signaling pathway |
| PLCE1 | hsa00562:Inositol phosphate metabolism,hsa04020:Calcium signaling pathway,hsa04070:Phosphatidylinositol signaling system |
| ZIC2 | hsa04340:Hedgehog signaling pathway |
| BTC | hsa04012:ErbB signaling pathway |
| ZMAT3 | hsa04115:p53 signaling pathway |
